# Biallelic loss of function NEK3 mutations deacetylate α-tubulin and downregulate NUP205 that predispose individuals to cilia-related abnormal cardiac left–right patterning

**DOI:** 10.1038/s41419-020-03214-1

**Published:** 2020-11-23

**Authors:** Yuan Zhang, Weicheng Chen, Weijia Zeng, Zhouping Lu, Xiangyu Zhou

**Affiliations:** 1 Department of Assisted Reproduction, and Clinical and Translational Research Center, Shanghai First Maternity and Infant Hospital, Tongji University School of Medicine, 201204 Shanghai, China; 2 Pediatric Cardiovascular Center, Children’s Hospital of Fudan University, 201102 Shanghai, China; 3 School of Life Sciences, Fudan University, 200433 Shanghai, China; 4 Clinical and Translational Research Center, Shanghai First Maternity and Infant Hospital, Tongji University School of Medicine, 201204 Shanghai, China

**Keywords:** Congenital heart defects, Genetics research

## Abstract

Defective left–right (LR) organization involving abnormalities in cilia ultrastructure causes laterality disorders including situs inversus (SI) and heterotaxy (Htx) with the prevalence approximately 1/10,000 births. In this study, we describe two unrelated family trios with abnormal cardiac LR patterning. Through whole-exome sequencing (WES), we identified compound heterozygous mutations (c.805-1G >C; p. Ile269GlnfsTer8/c.1117dupA; p.Thr373AsnfsTer19) (c.29T>C; p.Ile10Thr/c.356A>G; p.His119Arg) of *NEK3*, encoding a NIMA (never in mitosis A)-related kinase, in two affected individuals, respectively. Protein levels of NEK3 were abrogated in Patient-1 with biallelic loss-of function (LoF) *NEK3* mutations that causes premature stop codon. Subsequence transcriptome analysis revealed that NNMT (nicotinamide *N*-methyltransferase) and SIRT2 (sirtuin2) was upregulated by NEK3 knockdown in human retinal pigment epithelial (RPE) cells in vitro, which associates α-tubulin deacetylation by western blot and immunofluorescence. Transmission electron microscopy (TEM) analysis further identified defective ciliary ultrastructure in Patient-1. Furthermore, inner ring components of nuclear pore complex (NPC) including nucleoporin (NUP)205, NUP188, and NUP155 were significantly downregulated in NEK3-silenced cells. In conclusion, we identified biallelic mutations of NEK3 predispose individual to abnormal cardiac left–right patterning via SIRT2-mediated α-tubulin deacetylation and downregulation of inner ring nucleoporins. Our study suggested that NEK3 could be a candidate gene for human ciliopathies.

## Introduction

Left–right (LR) asymmetry is an essential aspect of embryonic development in most bilateral vertebrates and invertebrates^1^. Accurate positioning of asymmetric organs along the LR axis is crucial for their proper function, and defects in LR organization cause a range of malformations including situs inversus (SI) totalis (SIT) and heterotaxy (Htx)^2^. SIT is a congenital condition in which the organs in the chest and abdomen are arranged in a complete mirror-image reversal of the usual positions; the prevalence of SIT is estimated to range from 1/25,000 to 1/8000 (ref. ^3^). Up to 20% of patients with SIT have Kartagener syndrome (KS) which is a trilogy of symptoms (nasal polyps, bronchiectasis, and SIT). KS is a subgroup of primary ciliary dyskinesia (PCD)^4^. Approximately 50% of patients with PCD present with SIT, designated as KS^5^. KS and PCD are associated with the same respiratory symptoms that are secondary to ultrastructural anomalies of cilia^6^. Different from SIT, Htx is a condition that involves the internal organs being abnormally arranged but not in a mirror image within the chest and abdomen^7^. Over 80% of indiciduals with Htx present with complex congenital heart disease (CHD) including transposition of the great arteries and double outlet right ventricle^8^. SIT patients have a lower risk of CHD (3–9%) than patients with Htx. However, the risk of CDH in SIT is still much higher than in the normal situation, situs solitus (0.6–0.8%)^9^.

Previous studies have established the fundamental roles of embryonic cilia-driven fluid flow in LR organization in mammals^10,11^. Classified by microtubular structure, two types of cilia are found: primary/non-motile cilia and regular motile cilia. Most regular motile cilia consist of a ring of nine peripheral microtubule doublets that surrounds a central pair of single microtubules (9 + 2 structure). In contrast to the 9 + 2 pattern of regular cilia, primary or sensory cilia are solitary, immotile organelles that have 9 + 0 microtubule configuration and are present on most cell types^12^. Primary cilia are microtubule-based organelles involved in a variety of signaling cascades^13^. Of the many signaling pathways associated with primary cilia, NIMA (never in mitosis A)-related kinases (Nek1-Nek11), a family of Ser-Thr kinases, have been extensively illustrated in many cellular processes^14^. Although the functions of mammalian Nek proteins remain largely unknown, common themes in Nek kinases are cilia, centrosomes, and microtubule organization^15^. Nek2 is crucial for proper mitosis through its effects on centrosomes^16^, and Nek1 and Nek8 are involved in primary cilium formation^17,18^. Nek6 can phosphorylate the kinesin Eg5, which regulates microtubule movement^19^. Nek8 phosphorylates Bicd2, a protein that is important for microtubule maintenance and axonal stability^20^. A recent study identified NEK10 as a ciliated cell-specific kinase whose activity regulates the motile ciliary proteome to promote ciliary length and mucociliary transport^21^. More importantly, several Nek kinases have been implicated in embryonic LR determination. Rare genic copy number variations in NEK2 were identified in patients with Htx^22^. Nek2 is a key switch balancing cilia biogenesis that be required for normal LR patterning in *Xenopus*^23^. Nek8 homozygous null mice exhibit randomization of LR asymmetry^24^.

Different from most NIMA family members that closely associated with cell cycle regulation, inhibition of Nek3 activity did not affect cell cycle progression^25^. Two haplotypes have been identified in NEK3 that differ by a 1 nt indel (rs3837575, 0.541 in GnomAD) at the end of exon 10 (supplementary Fig. S1). Both haplotypes appear to encode functional proteins via alternative in-frame splicing of exon 11. In the full-length protein (NM_002498), the length of exon 10 is 73 bp. However, in the haplotype that lacking exon 11 (NM_001146099), the length of exon 10 is 72 bp. Expression of a phospho-defective mutant or truncated Nek3 leads to distorted neuronal morphology and deacetylation of microtubules via HDAC6 (ref. ^26^). In addition, NEK3 was reported to play important roles in cancer progression. NEK3 activates Rac1 and contributes to prolactin-mediated breast cancer motility through paxillin phosphorylation^27^. Phosphorylation of NEK3 at T165 modulates focal adhesion remodeling necessary for breast cancer cell migration^28^. In the following, by performing trio-based whole-exome sequencing (WES), we identified biallelic mutations in NEK3 in two unrelated patients with abnormal cardiac LR patterning and found NEK3 deficiency promotes NNMT expression and downregulates expression of inner ring components of NPC, which potentially associates with defective ultrastructure of cilia and abnormal cardiac LR organization.

## Materials and Methods

### Subjects

In this study, we collected two unrelated family trios with abnormal cardiac LR patterning. The affected individual in Family-1 is a 6-year-old boy who had been diagnosed as isolated dextrocardia at the age of 2 months by X-ray and color ultrasonic diagnosis at Pediatric Cardiovascular Center of the Children’s Hospital affiliated to Fudan University, Shanghai, China. In the Family-2, a 29-year-old female was diagnosed as SI totalis with mild scoliosis by X-ray at Pukou District Central Hospital, Nanjing, China. The fresh peripheral blood samples of patients and unaffected family members were collected for genomic DNA and RNA isolation, WES, and real-time PCR. For studies of affected individual and their families, written informed consent was obtained from all participants prior to the start of the study. All procedures in the study were approved by the Medical Ethics Committee of Children’s Hospital of Fudan University (2016-079) (Shanghai, China).

### Genetic analysis

WES was performed by utilizing the SureSelect human all exon platform (v.6; Agilent Technologies, Santa Clara, CA, USA). The Genome Analysis Toolkit software package (GATK4.1.2.0) was used for detection of single-nucleotide variants and indels. Variant filtering was performed according to minor allele frequencies (MAFs) under 0.01 in the GnomAD database (http://gnomad.broadinstitute.org/), given the rarity of situs abnormality and associations with protein-altering variants (missense, nonsense, and splice-site variants as well as coding indels). Recessive and homozygous variants were then maintained for subsequent analysis. The effects of the identified variants were assessed using SIFT (http://sift.jcvi.org), PolyPhen-2 (genetics.bwh.harvard.edu/pph2), and Mutationtaster (http://mutationtaster.org). Conservation analysis was performed using Multiple Sequence Alignment ClustalW2 (ebi.ac.uk/Tools/msa/clustalw2). Candidate causal variants of NEK3 were then confirmed by sanger sequencing and the sequences of primers are provided in Supplementary Table S1.

### cDNA analysis

Total RNA was isolated from fresh peripheral blood samples of the Patient-1 and his unaffected parents by using the RNAprep pure Blood Kit (DP433, TIANGEN) followed by first-strand cDNA synthesis (KR106, TIANGEN). cDNA was then amplified by polymerase chain reaction (PCR) with specific NEK3 primers followed by gel extraction (DP209, TIANGEN) and Sanger sequencing. The primers used in PCR reactions are listed in Supplementary Table S1.

### Cell culture

Human TERT retinal pigment epithelial (RPE) cells (ATCC®CRL-4000) were grown in Dulbecco’s modified Eagle’s medium (DMEM)/ F12 1:1: Liquid (HyClone) supplemented with 10% FBS (fetal bovine serum) (Gibco), 100 IU/ml penicillin, 100 μg/ml streptomycin at 37 °C in a 5% CO_2_ atmosphere.

### siRNA silencing

SiRNA reagent kits and the negative control were ordered from RiboBio Co. Ltd (Guangzhou, China). Three pairs of siRNAs (#1-3) against human *NEK3* (NM_002498) and two pairs of siRNAs (#1–2) against human *NUP205* (NM_015135) were designed for gene silencing and siRNA sequences are provided in Supplementary Table S2. Briefly, human RPE cells were grown in the presence of 10% FBS under identical culture conditions and were then transfected with Ctrl or each NEK3 or NUP205 siRNA (75 nM) using the LipofectAMINE 3000 reagent (Invitrogen, CA, USA) according to the standard protocols. At 60-h post-transfection, cells were collected to evaluate knockdown efficiency by western blot.

### RT-qPCR

Total RNA was isolated from Ctrl or NEK3-siRNA-treated human RPE cells using the RNAprep pure Cell Kit (DP430, Tiangen, Beijing, China) followed by first-strand cDNA synthesis. RT-qPCR was performed using the FastFire qPCR PreMix (SYBR Green) (FP207, TIANGEN) on an ABI StepOnePlus instrument. The delta-delta-Ct (ddCt) algorithm was utilized to analyze the relative changes in gene expression by using GAPDH as the internal reference (housekeeping gene). The data are presented as the mean ± SD of four independent experiments; each sample was assayed in triplicate in each experiment. RT-qPCR primer sequences are available in Supplementary Table S1 and were designed using Primer3Plus software to span exon–exon junctions.

### Western blot

siRNA-treated human RPE cells or nasal mucosa tissues of the patient were lysed on ice for 30 min in RIPA lysis buffer (Sangon Biotech) and lysates were clarified by centrifugation at 12,000 r.p.m. for 10 min at 4 °C. Western blotting was performed following standard procedures. Membranes were blocked with 5% non-fat milk for 1 h at room temperature and then incubated with different primary antibodies in 0.5% bovine serum albumin (BSA) or 5% milk. The bands on immunoblots were quantified using Image J software.

### Immunofluorescence

Human RPE cells were seeded on glass cover slips and grown in presence of 10% FBS under identical culture conditions. At 60-h post-transfection, cells were fixed and permeabilized for 10 min using 4% PFA and 0.8% Triton-X100. After blocking, cells were incubated with the primary antibody as aforementioned (anti-AcTub; Sigma-Aldrich, 1:500 dilution) overnight at 4 °C. Cells were then incubated with the secondary antibody (Alexa 488 anti-mouse; Thermo Fisher Scientific, 1:1000 dilution) for 2 h at room temperature, followed by staining with DAPI (Thermo Fisher Scientific)/PBS for 6 min. Confocal imaging was performed using an SP8 system (Leica), and images were processed with using the Leica AF software suite.

### High-speed video microscopy and TEM analysis

Nasal tissues of Patient-1 and the healthy individual were collected and maintained in L-15 medium (Invitrogen, CA) for video microscopy using a Leica inverted microscope (Leica DMI300B) as described previously^29,30^. Movies were recorded at 200 frames/s at room temperature by use of a 680 PROSILICA GE camera (Allied Vision, PA). Transmission electron microscopy (TEM) analysis was performed at the Department of Electron Microscopy, Fudan University as previously^29,30^. Briefly, the samples were fixed with glutaraldehyde (2.5% w/v) in 0.1 M sodium cacodylate buffer. Following fixation in osmium tetroxide (1% w/v), samples were dehydrated through graded ethanol series and embedded in epoxy resin. Sections were cut at 50–70 nm thickness and stained with 2% methanolic uranyl acetate and Reynold’s lead citrate for analysis by using a TEM (JEM-1400; Jeol, Tokyo, Japan).

### RNA sequencing

Transcriptome libraries were prepared using a TruSeq RNA Sample Preparation kit (Illumina) and then sequenced using the Illumina HiSeq PE platform at 2 × 151-bp read lengths. The expression level of each transcript was calculated according to fragments per kilobase of exon per million mapped reads (FPKM). Differentially expressed genes (DEGs) between the different groups were selected based on a log fold-change greater than 2 and a false-discovery rate of less than 0.05. Gene ontology (GO) functional-enrichment and Kyoto Encyclopedia of Genes and Genomes pathway analyses were performed using GOATOLLS and KOBAS. DEGs were considered significantly enriched when the *p* value was less than 0.05 following Bonferroni correction.

### Antibodies

For immunoblots, proteins were probed with one of the following primary antibodies in either 5% non-fat milk or 5% bovine serum albumin: anti-HEC1 (#3613, 1:1000 dilution) and anti-NEK3 (#37636, 1:800 dilution) antibodies from Abcam; anti-XPO1 (#46249, 1:1000 dilution), anti-phospho-Akt (Ser473) (#4051, 1:1000 dilution), and anti-GAPDH (#5174, 1:3000 dilution) from Cell Signaling Technology; anti-NUP205 (Santa Cruz Biotechnology, 1:200 dilution), and anti-tubulin (acetylated) (T6793, Sigma-Aldrich, 1:1000 dilution for WB).

### Statistics

The data are presented as the mean ± standard. Comparisons between groups were analyzed with two-tailed nonparametric Mann–Whitney test by using statistical software SPSS version 16 (SPSS Inc., Chicago, USA). A *p* value of less than 0.05 was considered statistically significant.

## Results

### Case presentation

**Fig. 1:**
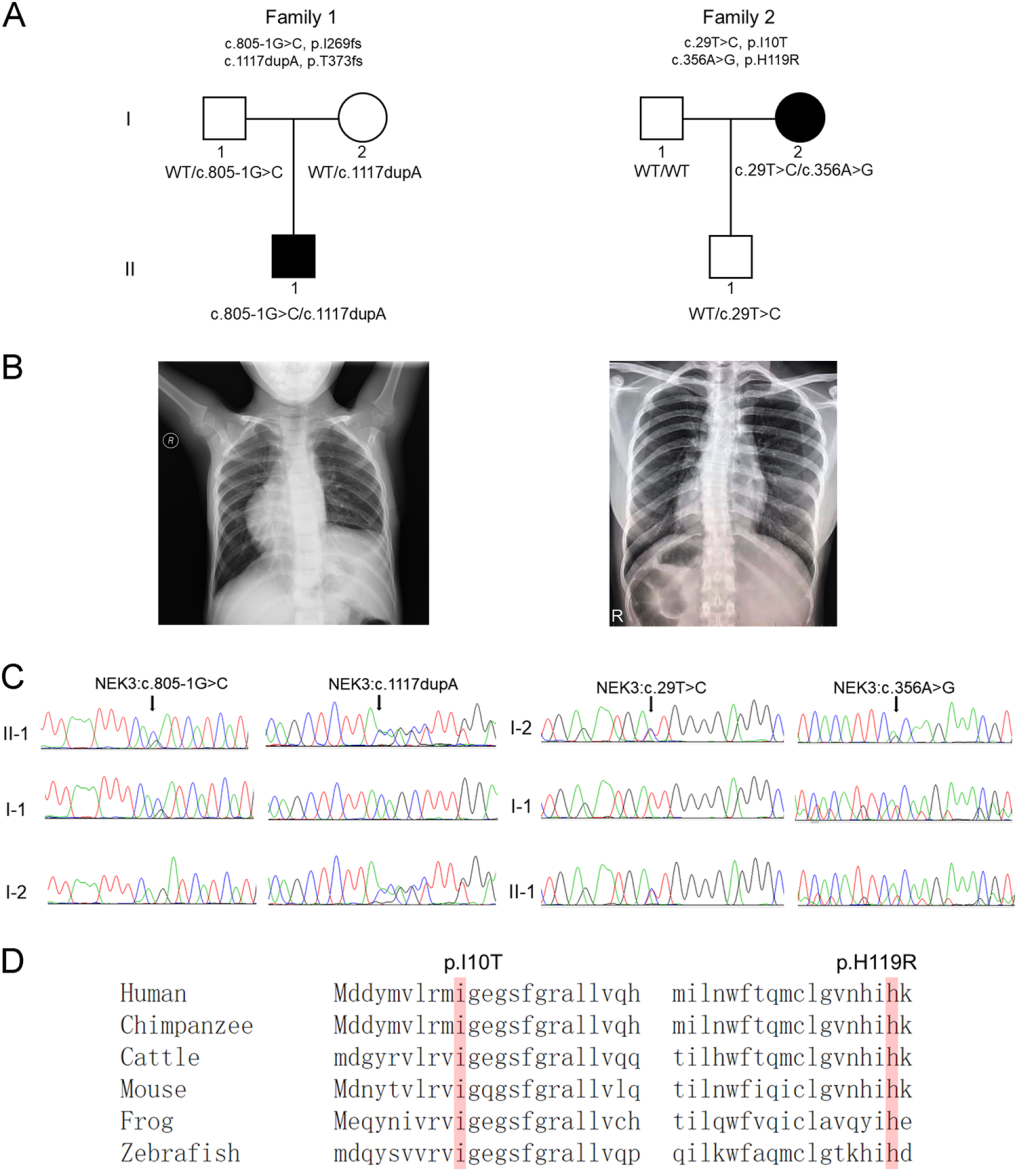
Biallelic NEK3 mutations in two unrelated patients with abnormal LR patterning. **a** Pedigree of Family-1 and -2 indicating the affected individuals and the segregation of NEK3 recessive variants. **b** Chest X-ray shows isolated dextrocardia and raised diaphragm on the left side in the Patient-1 (left), and the mirror-image arrangement of the abdominal organs and mild scoliosis in the Patient-2 (right). **c** Sanger segregation of NEK3 variants (c.805-1G>C/c.1117dupA; c.29T>C/c.356A>G) in the patients and unaffected individuals as indicated. **d** Sequencing alignment of missense variants p.I10T (c.29T>C) and p.H119R (c.356A>G) in different species including zebrafish (*Danio rerio*) and tropical clawed frog (*Xenopus tropicalis*).

In this study, we collected two unrelated family trios with abnormal cardiac LR patterning. The affected individual in Family-1 is a 6-year-old boy of Chinese origin; both his parents were unaffected (Fig. 1a). Chest X-ray showed isolated dextrocardia and raised left side of the diaphragm in this boy (Fig. 1b). Echocardiography did not reveal other detectable abnormalities in heart structure or motion. Typical PCD phenotypes including bronchitis, sinusitis, and otitis media were temporarily excluded by physical examination as previously^29,30^. In the Family-2, the patient is a 29-year-old female; her husband and 3-year-old son were healthy (Fig. 1a). Chest X-ray showed the mirror-image arrangement of the abdominal organs including heart and liver in the patient (Fig. 1b). She exhibited normal growth parameters without mental retardation and could withstand some high-intensity exercises like mountain-climbing. Other abnormalities in the heart, lung, and liver were excluded by physical examination. The Patient-2 had no clinical symptoms of PCD including bronchitis, sinusitis, and otitis media according to her complaint in life history. Chromosomal microarray analysis did not detect potential pathogenic copy number variations in these two affected individuals.

### Biallelic mutations in NEK3 in two patients with defective cardiac LR patterning

Through trio-based WES analysis, we identified compound heterozygous non-synonymous mutations (c.805-1G>C/c.1117dupA; c.29T>C/c.356A>G) of *NEK3* (NM_002498) (Fig. 1c), encoding a NIMA (never in mitosis A)-related kinase, in two patients. In the patient-1, the MAF (MAF) were 0.00068 for paternally derived c.805-1G>C and 0.00047 for maternally derived c.1117dupA (Table 1). According to GnomAD database, c.805-1G>C and c.1117dupA (p. Thr373fs) were annotated as splicing acceptor site and frameshift variants, respectively. In the patient-2, the MAF were 0.00009 for c.29T>C (p.Ile10Thr) and 0.00048 for c.356A> G (p.His119Arg) (Table 1). These two missense variants p.Ile10Thr and p.His119R were highly conserved in different species including Zebrafish (*Danio rerio*) and Tropical clawed frog (*Xenopus tropicalis*) (Fig. 1d), and were uniformly predicted to be damaging by Poly-phen2, SIFT and Mutationtaster (Table 1).

**Table 1:** Biallelic NEK3 mutations in two unrelated individuals with defective LR patterning.

	Affected individual	Gene	Variant information	Poly-phen2	Mutation-Taster	SIFT	MAF in GnomAD
Family-1	Male, 6s	NEK3	c.805-1G>C; p.Ile269GlnfsTer8 c.1117dupA; p.Thr373AsnfsTer19	LoF LoF	LoF LoF	LoF LoF	0.000683 0.000474
Family-2	Female, 29s	NEK3	c.29T>C; p.Ile10Thr c.356A>G; p.His119Arg	0.996-D 1.000-D	0.001-D 0.001-D	0.999-D 0.999-D	0.000099 0.000489

*LoF* loss of function (stop-gain/frameshift/splicing site), *D* damaging, *MAF* minor allele frequency.

### c.805-1G>C leads to exon 10 skipping and generates premature translation termination

**Fig. 2:**
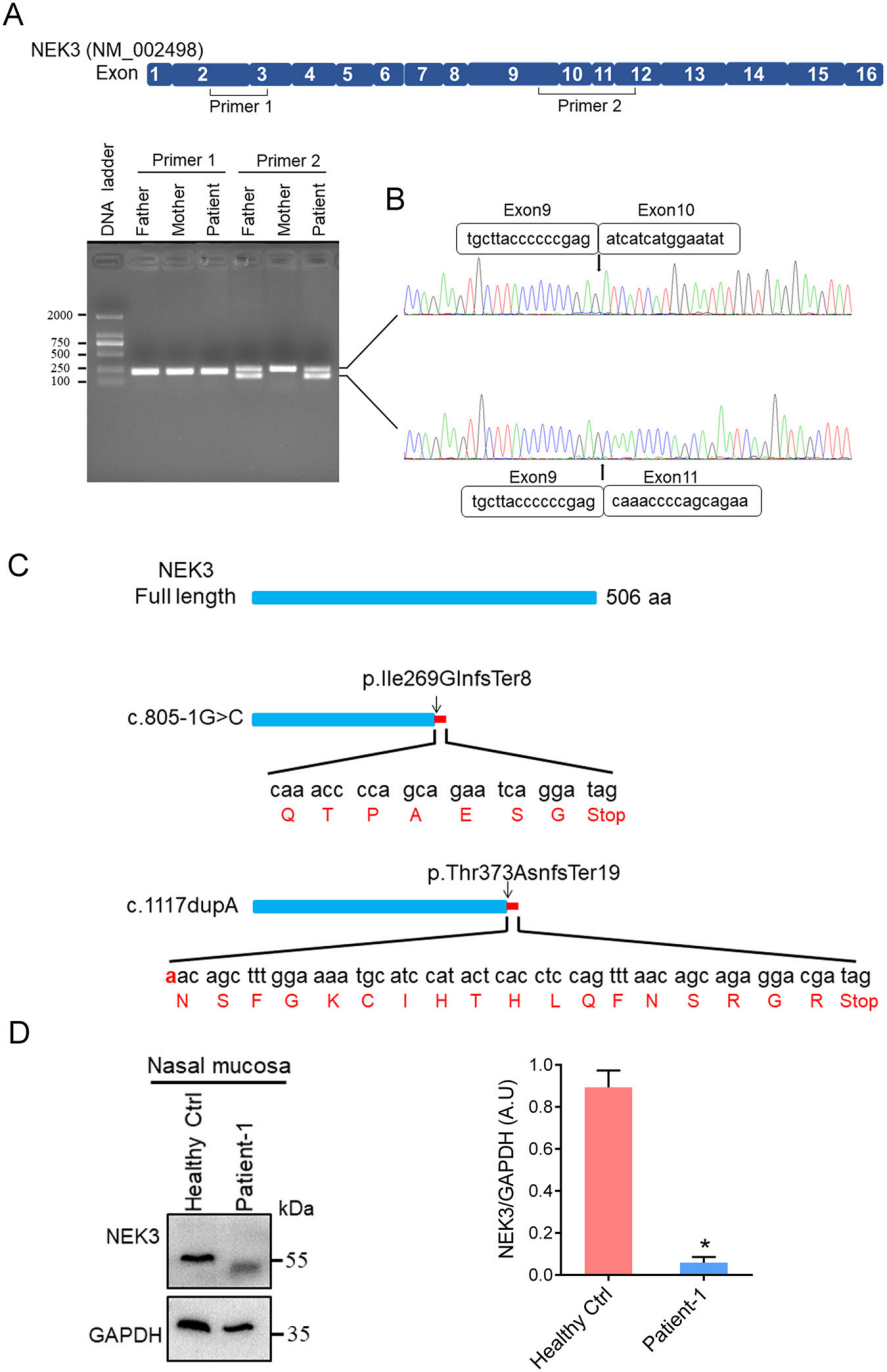
NEK3 c.805-1G>C causes exon-10 skipping and premature termination of translation. **a** Amplification of cDNA derived from fresh blood samples of the patient and his parents by using two different pairs of primers (primer-1 and -2) as indicated. **b** Sanger sequencing demonstrated that exon 10 (73-bp) was skipped in the smaller band in the patient and his father carrying c.805-1G>C when using primer-2. **c** Compared with full-length protein (506 amino acids), both NEK3 c.805-1G>C (p. Ile269GlnfsTer8) and c.1117dupA (p. Thr373AsnfsTer19) lead to premature termination of translation. **d** Protein levels of NEK3 in the nasal mucosa of Patient-1 and healthy individual were evaluated by western blot. GAPDH served as a loading control. Right panel: Quantification data of NEK3 expression level when normalized against GAPDH. *p value <0.05 (Mann–Whitney *U* test). AU arbitrary units. Representative image from three independent experiments was displayed.

To study the effects of splicing acceptor site variant c.805-1G>C on mRNA splicing, we performed real-time PCR using cDNAs derived from fresh peripheral blood samples of patient and his unaffected parents of Family-1. Two different pairs of primers were designed for PCR amplification as indicated (Fig. 2a). We did not identify the difference on the size of PCR products when using Primer-1. However, a smaller size of PCR band that uniquely exists in the cDNA samples of patient and his father that carrying c.805-1G>C was identified when using Primer-2 (Fig. 2b). Sanger sequencing further demonstrated that exon 10 (73-bp length) of NEK3 was entirely skipped in this smaller band, which would induce an out-of-frame alteration. Thus, both c.805-1G>C (p.Ile269GlnfsTer8) and c.1117dupA (p.Thr373AsnfsTer19) could generate prematurely terminated protein translation (Fig. 2c). As a consequence, protein level of NEK3 were almost abrogated in the nasal mucosa of Patient-1 when comparing with a health male (Fig. 2d).

### NEK3 deficiency promotes expression of NNMT and SIRT2, and deacetylates α-tubulin

**Fig. 3:**
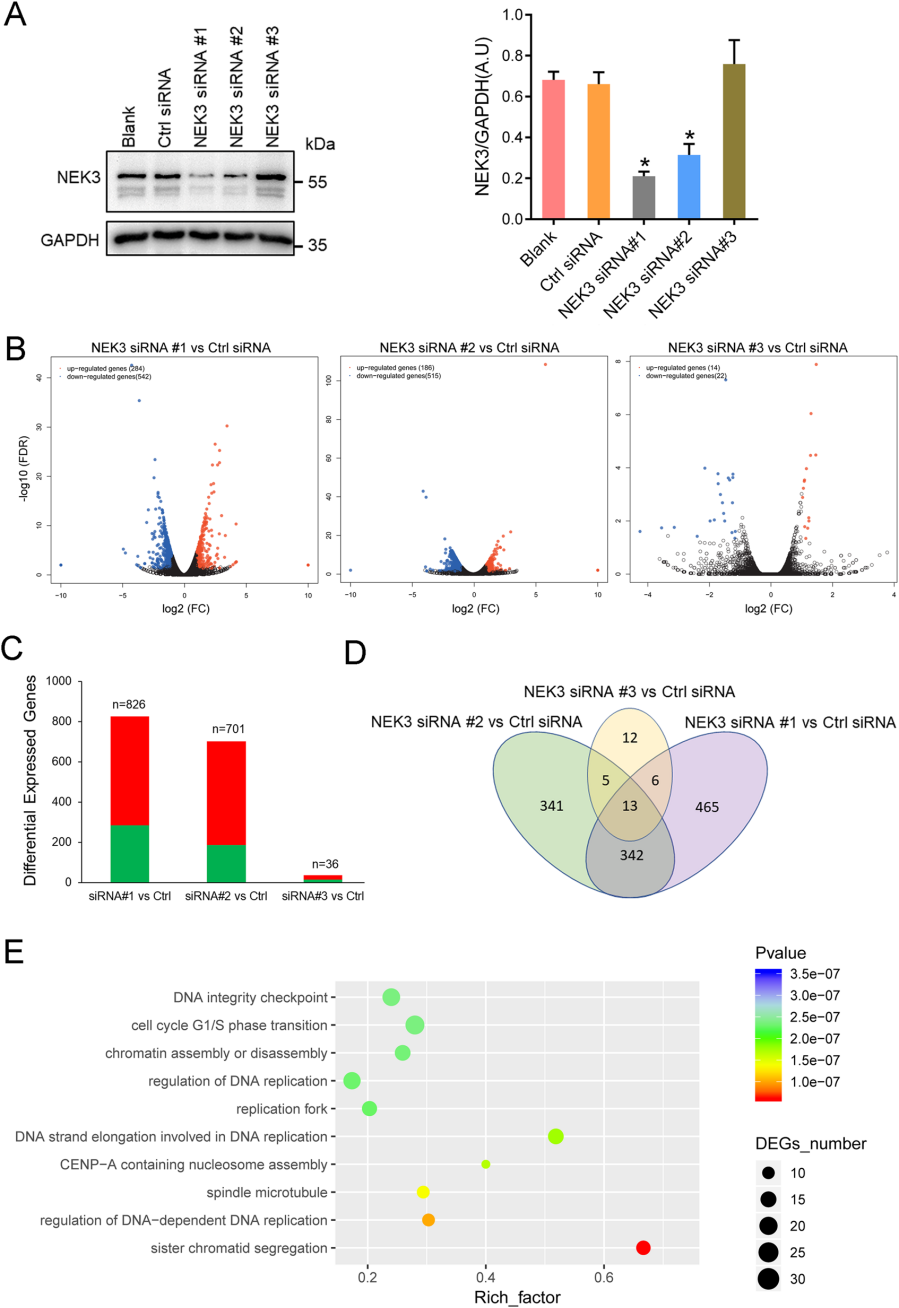
NEK3 deficiency interferes chromosome segregation, DNA replication, and spindle microtubule. **a** Evaluation of the efficiencies of three siRNAs targeting NEK3 in human RPE cells by western blots. Right panel: Quantification data of NEK3 expression level when normalized against GAPDH. *p value<0.05 (Mann–Whitney *U* test). AU arbitrary units. Representative image from three independent experiments was displayed. **b** Volcano plots showing the differentially expressed genes (DEGs) (*p* value <0.05; fold-change>2) for each group. Up- and downregulated genes are highlighted red and blue, respectively. **c** Numbers of DEGs were displayed. Up- and downregulated genes are highlighted red and green, respectively. **d** Overlap of DEGs between different groups as indicated. **e** GO enrichment analysis of 342 D**E**Gs revealed that chromatid segregation, DNA replication, and spindle microtubule were the top 3 enriched terms.

To mimic the biological consequences of *NEK3* biallelic loss-of-function (LoF) mutations on cellular biological processes, we knocked down the expression of *NEK3* by using small interfering RNAs (siRNAs). Human TERT RPE cells were transfected with control (Ctrl) or each NEK3 siRNA#1-3. Silencing efficiency was tested by western blots showing that siRNAs #1 and #2 largely depleted NEK3 protein levels (Fig. 3a), whereas siRNA #3 and Ctrl siRNA had no significant effect on *NEK3* silencing. Subsequent RNA-seq based transcriptome analysis identified 826 and 701 DEGs in NEK3-silenced hRPE cells mediated by siRNAs #1 and #2, respectively, whereas 36 DEGs were identified in cells transfected with siRNA #3 compared with Ctrl siRNA (Fig. 3b, c). After exclusion of unspecific DEGs generated by Ctrl siRNA or ineffective siRNA#3, a total of 342 DEGs were specially shared by siRNA #1 and #2 mediated NEK3 silencing (Fig. 3d). GO enrichment analysis of these 342 DEGs revealed that sister chromatid segregation (*p* = 6.19E−8), DNA replication (*p* = 1.01E−7), and spindle microtubule (*p* = 1.38E−7) were the top 3 enriched terms (Fig. 3e). This finding suggested NEK3 deficiency might interfere microtubule assembly.

**Fig. 4:**
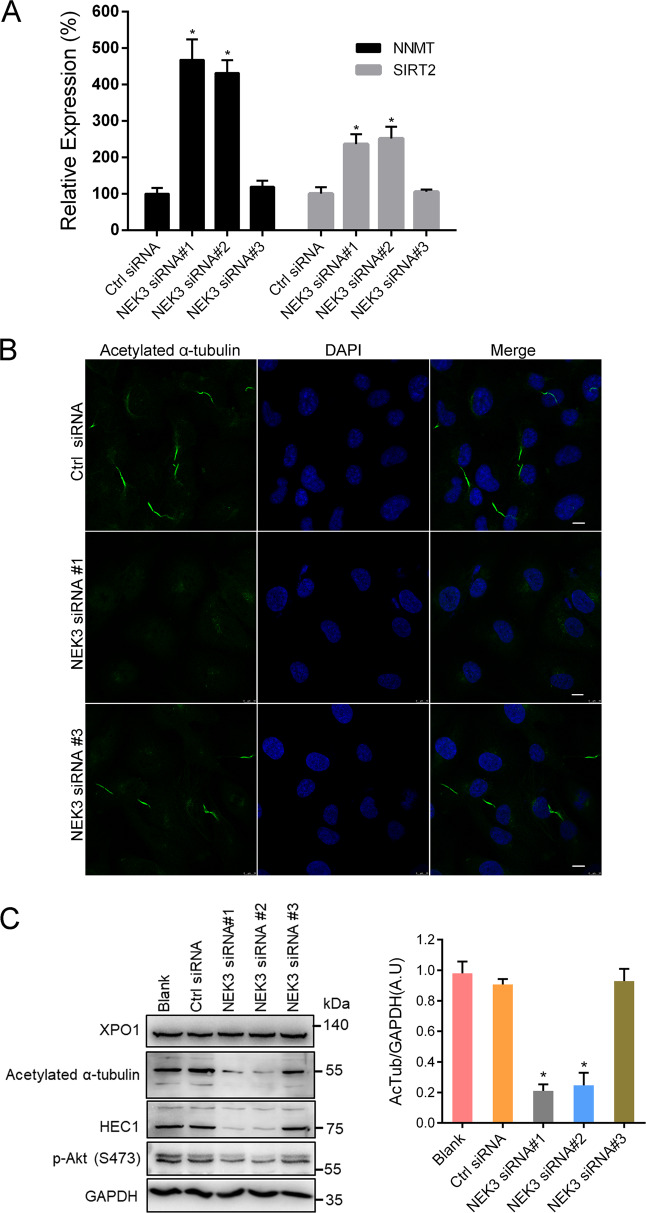
NEK3 deficiency promotes expression of NNMT and SIRT2, and deacetylates α-tubulin. **a** Upregulation of NNMT and SIRT2 in NEK3-silenced cells were validated by real-time quantitative PCR. *P value <0.05 (Mann–Whitney *U* test, *n* = 4). **b** Immunofluorescence assay by staining acetylated α-tubulin antibody using a confocal microscope in RPE cells transfected with Ctrl siRNA, NEK3 siRNA#1 or ineffective siRNA#3 in vitro. Scale bar, 10 μm. **c** Protein expression of acetylated-ɑ-tubulin, HEC1, XPO1, p-AKT(S473) in NEK3-silenced RPE cells was evaluated by western blot. GAPDH served as a loading control. Right panel: Quantification data of acetylated-ɑ-tubulin (AcTub) protein level when normalized against GAPDH. *p value <0.05 (Mann–Whitney *U* test). AU arbitrary units. Representative image from three independent experiments was displayed.

In this study, mRNA levels of both NNMT (nicotinamide *N*-methyltransferase) and Sirt2 (sirtuin2) were significantly upregulated in the NEK3-silenced cells by transcriptome analysis followed by qPCR validation (Fig. 4a). Previous study found expression of NNMT in human neuroblastoma cells significantly increased the expression of Sirt1/2/3 (ref. ^31^). Sirt2 activity could be inhibited by the substrate of NNMT, nicotinamide. Sirt2 is the major effector that involving the α-tubulin deacetylation and regulates cilia length^32,33^. Acetylated microtubules have been considered to be stable, long-lived microtubules and α-tubulin deacetylation is associated with cilia resorption or disassembly^34^. In addition, a well-defined ciliary protein KIF7 that controls cilium architecture^35–37^ was significantly downregulated by NEK3 knockdown according to transcriptome analysis (Supplementary Table S2). Therefore, we performed immunofluorescence staining for acetylated α-tubulin. By using a confocal microscope, we observed that cilia biogenesis was disrupted by NEK3 knockdown mediated by both siRNA #1 and #2 whereas siRNA#3 had no effect on this process when compared with Ctrl siRNA in human RPE cells. Representative images of Ctrl siRNA, NEK3 siRNA#1, and siRNA#3 were displayed in Fig. 4b. Western blot further confirmed the markedly downregulation of acetylated α-tubulin and HEC1, the main component of the NDC80 kinetochore complex, in NEK3-silenced RPE cells whereas XPO1 and phosphor-Akt (Ser473) were not significantly affected (Fig. 4c).

### Defective ciliary ultrastructure in Patient-1 with NEK3 mutations

**Fig. 5:**
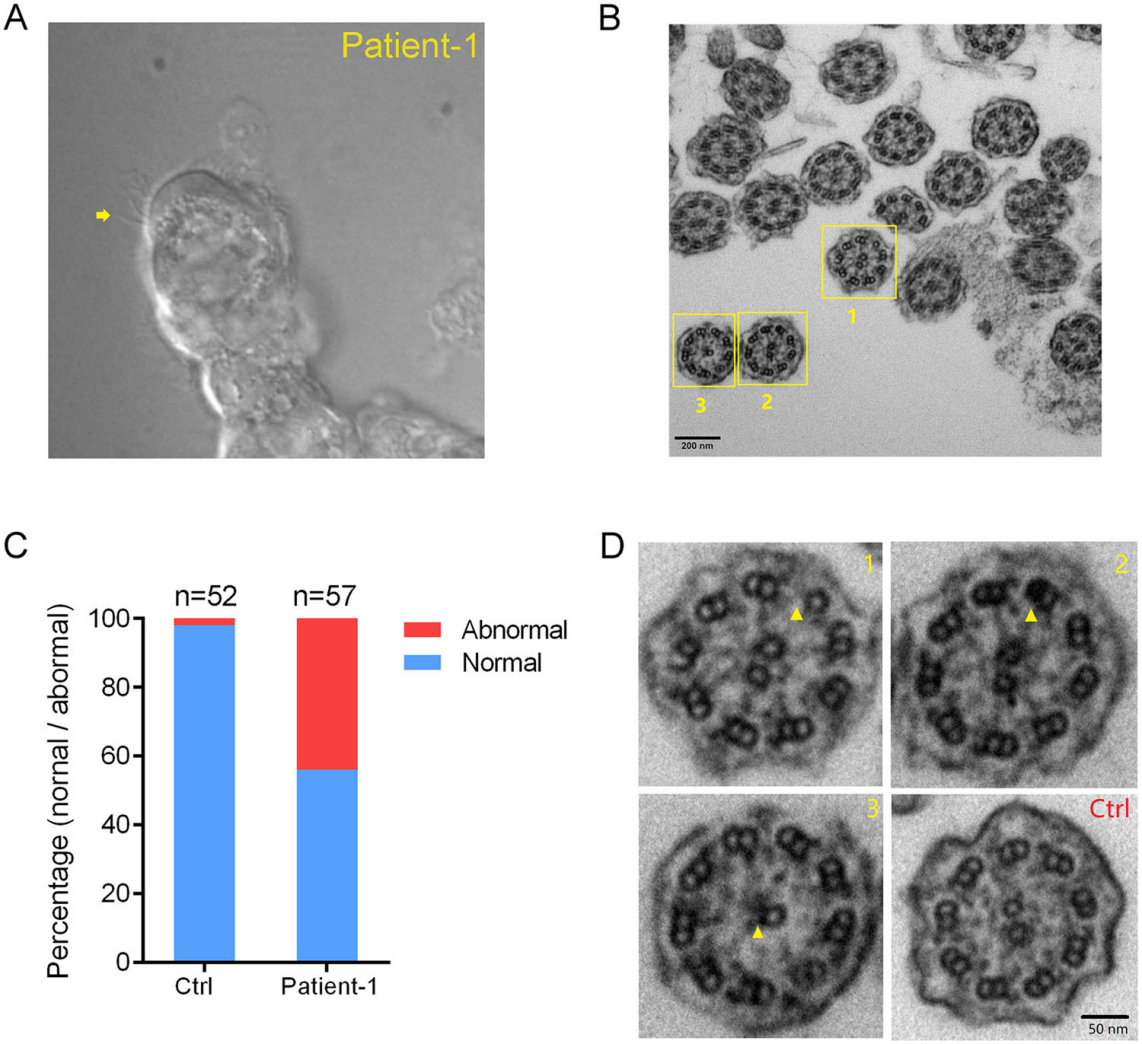
Defective ciliary ultrastructure in Patient-1 with NEK3 biallelic mutations. b Representative image showing primary nasal respiratory epithelial cells of Patient-1 by an optical microscope. Paucity of cilia was observed and indicated by yellow arrow. **b** Representative image of transmission electron micrographs (TEM) of ciliary axonemes in cross-section from Patient-1. Scale bar, 200 nm. **c** Percentage of abnormal ciliary ultrastructures in the Patient-1 and healthy individual. **d** Compared with a healthy individual (Ctrl), multiple ultrastructural abnormalities (#1–3) including the absence of the central-pair microtubules and nine peripheral microtubule doublets were identified in the Patient-1 that highlighted by yellow arrowheads. Scale bar, 50 nm.

To study the nature of NEK3-associated structural defects, we established culture of primary human nasal respiratory epithelial cells and identified restricted ciliary motion with shorten forward and recovery stroke in Patient-1 as previously (29,306) (Fig. 5a) We next assessed by TEM the ultrastructural effect of NEK3 mutations on cilia from the nasal mucosa of Patient-1 (Fig. 5b). Overall, we examined >50 cilia cross-sections without bias toward measurements in any particular cilia region. the percentage of abnormal cilia is 43.8% (25/57) in Patient-1 and 1.92% (1/52) in the healthy individual (Fig. 5c). Ultrastructural analyses identified multiple ultrastructural abnormalities including the absence of the central-pair microtubules and nine peripheral microtubule doublets when compared to a healthy male (Fig. 5d). These findings indicated that NEK3 mutations are associated with the defective microtubule assembly and ultrastructural abnormalities in the Patient-1.

### NEK3 deficiency downregulates mRNA levels of main components of inner rings of NPC

Inner ring complex of the NPC was constituted by Nup93, Nup205, Nup188, and Nup155 (ref. ^38^). Inner ring components of the NPC were essential for nuclear assembly and nuclear envelope formation^39,40^. A recent study found inner ring components of the NPC, but not other nucleoporins, specially localize and function at the cilium base^41^. Depletion of Nup188 and its binding partner Nup93 leads to a global defect in LR patterning in *Xenopus*^41^. Moreover, we previously identified biallelic NUP205 mutations in patients with abnormal cardiac LR patterning and depletion of Nup205 cause abnormal cardiac LR organization in Zebrafish^42^. In addition, inner ring components of the NPC were essential for nuclear assembly and nuclear envelope formation^43,44^.

**Fig. 6:**
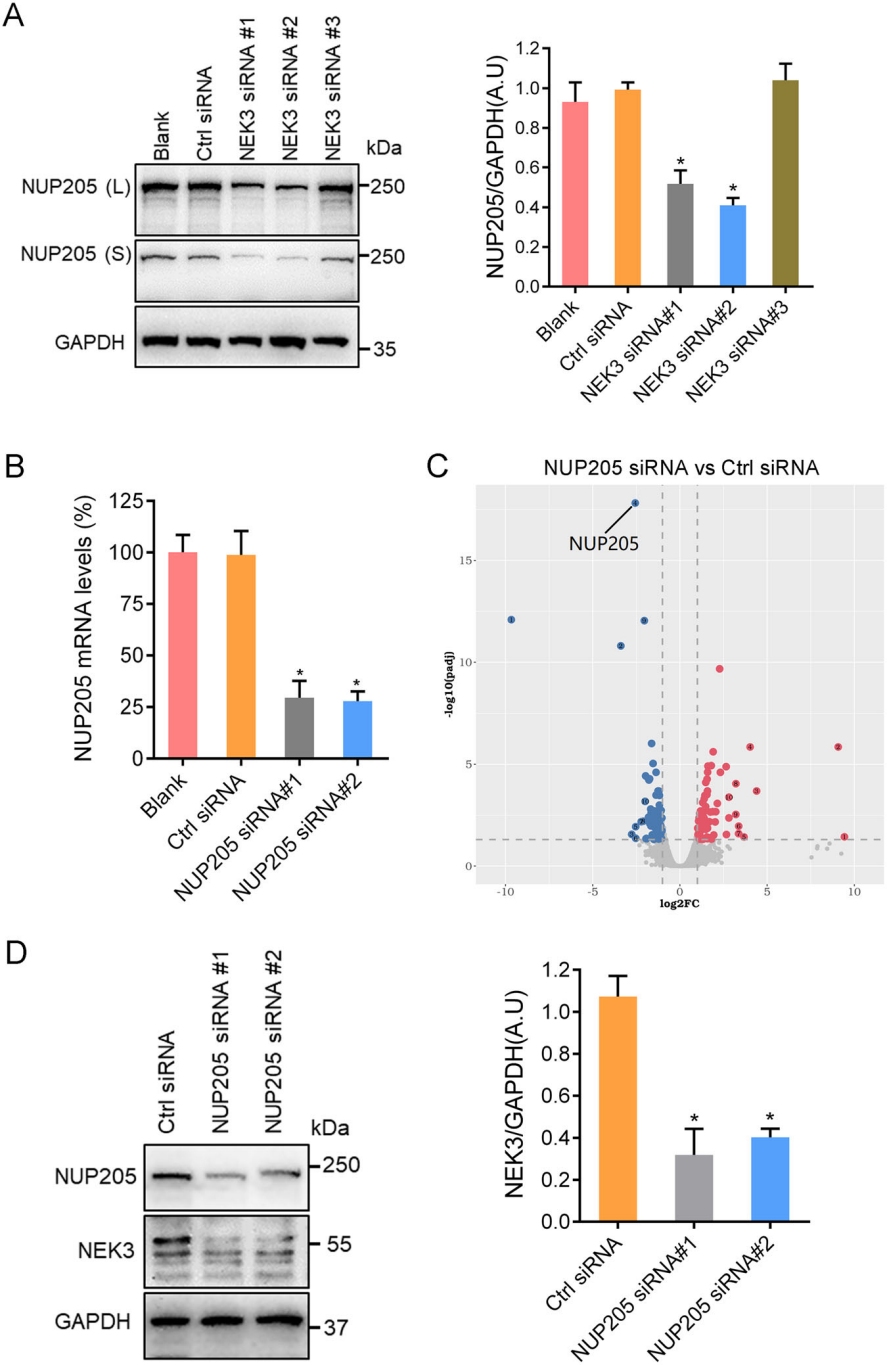
Reciprocal regulatory interactions between the NEK3 and NUP205. **a** Western blot showed protein level of NUP205 was downregulated in the NEK3-silenced cells. L long exposure, S short exposure. Right panel: Quantification data of NUP205 expression level when normalized against GAPDH. *P value<0.05 (Mann–Whitney *U* test). Representative image from three independent experiments was displayed. **b** Evaluation of the efficiencies of two siRNAs targeting NUP205 in human RPE cells by real-time qPCR. *p value <0.05 (Mann–Whitney *U* test, *n* = 4). **c** Volcano plots of DEGs (*p* value <0.05; fold-change>2) by NUP205 siRNAs mediated silencing in RPE cells when compared with Ctrl siRNA. Up- and downregulated genes are highlighted red and blue, respectively. The plot indicating downregulated NUP205 was highlighted by black line. **d** Western blot showed that protein levels of NEK3 was compromised by NUP205 knockdown in human RPE cells. GAPDH served as a loading control. Right panel: Quantification data of NEK3 expression level when normalized against GAPDH. *P value < 0.05 (Mann–Whitney *U* test). AU arbitrary units. Representative image from three independent experiments was displayed.

In this study, transcriptome analysis revealed that three components of inner ring of NPC, including NUP205, NUP188, and NUP155 were significantly downregulated in the NEK3-silenced cells (Supplementary Table S3), which explains that chromatin assembly or disassembly pathway was enriched in GO analysis (*p* = 2.34E−7). Downregulation of NUP205 is further confirmed by western blot (Fig. 6a). To assess the effects of NUP205 deficiency on cellular biological processes, we explored siRNA-mediated NUP205 knockdown in hRPE cells and performed transcriptome analysis. Silencing efficiency was tested by real-time qPCR showing that both two siRNAs #1 and #2 effectively knockdown NUP205 expression (Fig. 6b). Very interestingly, RNA-seq based transcriptome analysis revealed that NEK3 is significantly downregulated in NUP205-silenced cells (Fig. 6c) followed by western blot validation (Fig. 6d). This finding implicates the potential reciprocal regulatory interactions between the NEK3 and NUP205 on gene expression.

## Discussion

Abnormalities in cilia ultrastructure lead to a range of human phenotypes termed as ciliopathies, which include nephronophthisis, respiratory infection, infertility, retinal degeneration, situs abnormality, and skeletal dysplasia^45^. Nek kinases were initially studied for their roles in regulating cilia, centrosomes, and microtubule dynamics during cell cycle. Many NEKs including NEK1/2/8/9/10 have found to be mutated in human ciliopathies^21,22,46–48^. Here, we firstly identified biallelic LoF mutations of NEK3 in two unrelated patients with cilia-related abnormal cardiac LR patterning. Previous study found that NEK3 was potentially involved in actin polymerization by regulating the levels of F-action (polymerized form), which is essential for prolactin-mediated cytoskeletal reorganization^27^.

NEK3 have two functional alleles in humans via alternative in-frame splicing of exon 11. In the full-length allele-1 (NM_002498), c.805-1G>C would induce an out-of-frame deletion as shown in the Patient-1. However, in the shortened allele-2 (NM_001146099) in which the length of exon 10 is 72 bp, c.805-1G>C have no effect on the reading frame and just induce a non-frameshift deletion, which possibly still expresses a functional protein. This finding suggests the genetic consequence of the c.805-1G>C of NEK3 on disease occurrence could be altered in different allele.

HDAC6 and SIRT2 were two major effectors that deacetylates α-tubulin^32,49^. Previous studies have found that Nek3 regulates microtubule via HDAC6 acetylation in neurons involving axonal degeneration^26^. Given the similarities between cilia and neuronal axons (both are microtubule-rich structures extending from the cell body), we initiated studies and found cilia biogenesis in human RPE cell was disrupted by NEK3 deficiency via SIRT2 deacetylation. In our study, mRNA levels of HDAC6 and other HDAC members were not significantly changed in NEK3-silenced cells. Intriguingly, SIRT2 ablation was reported to have no effect on tubulin acetylation in brain or the progression of Huntington’s disease phenotypes^50^. These findings further suggested SIRT2 and HDAC6 exhibited potential tissue specificity on microtubule deacetylation. Moreover, overexpression of SIRT2 in renal epithelial cells was reported to cause decreased cilia length and morpholino knockdown of SIRT2 increased cilia length in Zebrafish^51,52^, which implicates the regulatory role of SIRT2 on cilia formation.

In this study, NNMT was the one of most upregulated DEGs in NEK3-silenced cells. NNMT catalyzes the reaction between nicotinamide and *S*-adenosylmethionine to produce 1-methylnicotinamide and S-adenosylhomocysteine. NNMT could promote the expression and stability of SIRTs^31,53^, and be inhibited by substrate of NNMT, nicotinamide^51^. These findings suggested upregulated NNMT might promote the expression of SIRT2 in the NEK3-silenced cells. In addition, NNMT is overexpressed in various human tumors and involved in the development and progression of several carcinomas^54^.

Both mRNA and protein levels of Hec1 were markedly downregulated in the NEK3-depleted cells. The outer kinetochore complex component Ndc80/Hec1 plays a critical role in regulating microtubule attachment to the spindle for accurate sister chromatid segregation^55,56^. GO analysis demonstrated that sister chromatid segregation is the most enriched term by DEGs of transcriptome analysis in our study. Very interestingly, a controversial model was recently proposed that biased chromatid segregation was involved in the LR symmetry breaking in mice without directly involving the Nodal gene^57^.

We identified the potential reciprocal regulatory interactions between the NEK3 and NUP205. Previous study found phosphorylation of NUP98 by multiple kinases, mainly NEK2/6/7, is crucial for NPC disassembly during mitotic entry^58^. Thus, investigating the effect of NEK3 site-specific point mutation on phosphorylation of inner ring components of the NPC like NUP205 and NUP188 are a potential avenue for further mechanistic understanding of these observations. Overall, our study shows that recessive variants in NEK3 are associated with abnormal cardiac LR patterning. Meanwhile, NEK3 could be a candidate gene for human ciliopathies.

## Supplementary information

Supplementary Figure and Table Legends

Supplementary Figure S1

Supplementary Table S1

Supplementary Table S2

Supplementary Table S3

## Data Availability

Raw sequencing data have been deposited in the NCBI Sequence Read Archive (SRA) database under accession number SRP281163. Other data that support the findings of this study are available from the corresponding authors upon reasonable request.
