# Supplementary material for: Biallelic loss of function NEK3 mutations deacetylate α-tubulin and downregulate NUP205 that predispose individuals to cilia-related abnormal cardiac left–right patterning
Source: Cell Death Dis. 2020 Nov 23;11(11):1005. doi: 10.1038/s41419-020-03214-1 (PMC7684299; doi:10.1038/s41419-020-03214-1)
Supplement: Supplementary file 1 — Supplementary Figure and Table Legends [file 41419_2020_3214_MOESM1_ESM.docx]

**Supplementary Figure and Table Legends**

Figure S1. Two haplotypes (Hap-1/-2) of NEK3 in the human population that differ by a 1 nt indel (rs3837575) at the end of exon 10. Both haplotypes appear to encode functional proteins via alternative splicing of exon 11.

Table S1. Sequences of primers and siRNAs used in this study.

Table S2. Ciliary protein KIF7 was significantly downregulated by NEK3 knockdown by transcriptome analysis.

Table S3. Nucleoporins expression in NEK3-silenced cells by transcriptome analysis.
