# Supplementary figures and images for: Biallelic loss of function NEK3 mutations deacetylate α-tubulin and downregulate NUP205 that predispose individuals to cilia-related abnormal cardiac left–right patterning
Source: Cell Death Dis. 2020 Nov 23;11(11):1005. doi: 10.1038/s41419-020-03214-1 (PMC7684299; doi:10.1038/s41419-020-03214-1)

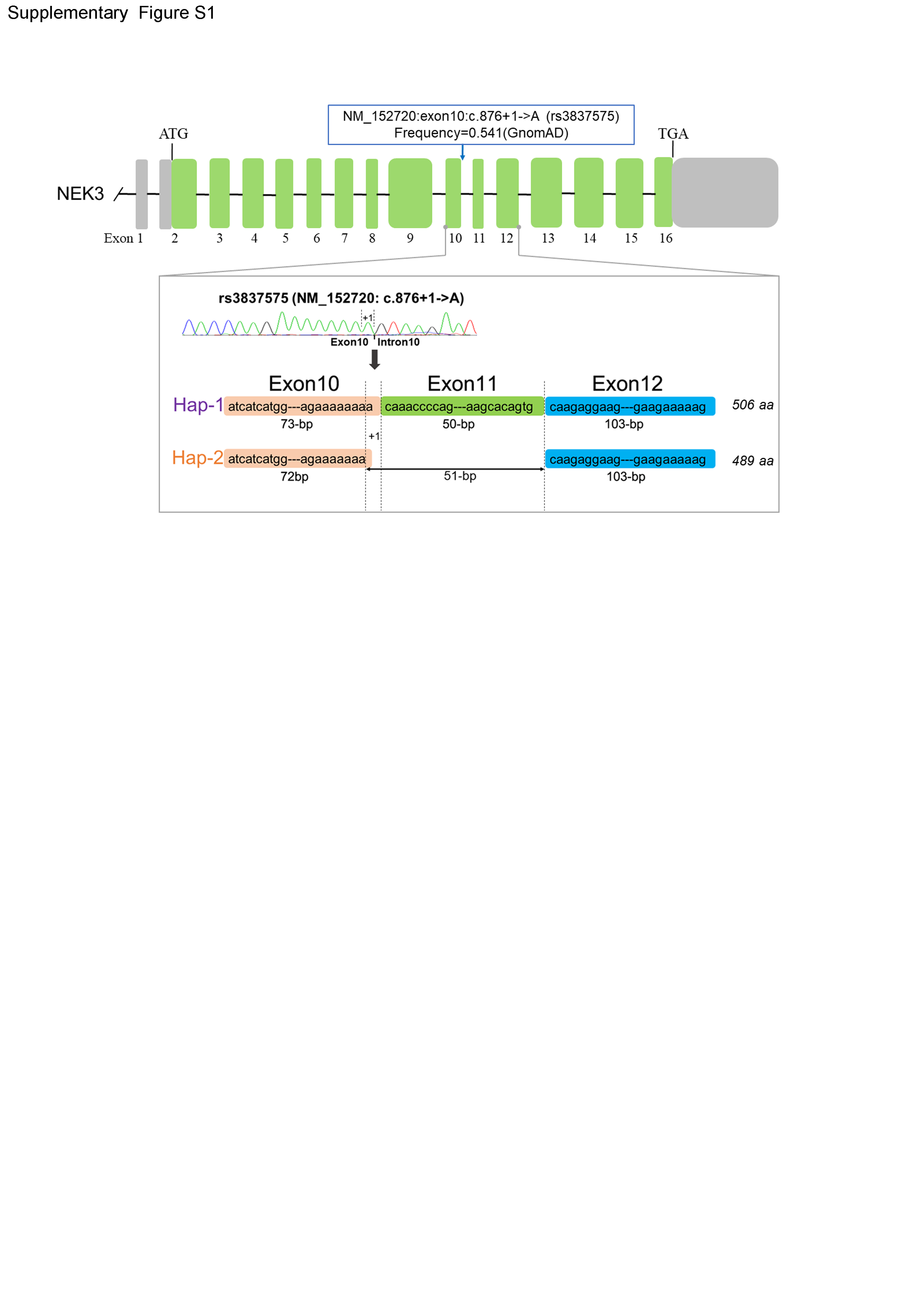

Supplement: Supplementary file 2 — Supplementary Figure S1 [file 41419_2020_3214_MOESM2_ESM.tif]
