# Supplementary material for: Biallelic loss of function NEK3 mutations deacetylate α-tubulin and downregulate NUP205 that predispose individuals to cilia-related abnormal cardiac left–right patterning
Source: Cell Death Dis. 2020 Nov 23;11(11):1005. doi: 10.1038/s41419-020-03214-1 (PMC7684299; doi:10.1038/s41419-020-03214-1)
Supplement: Supplementary file 3 — Supplementary Table S1 [file 41419_2020_3214_MOESM3_ESM.docx]

Supplementary Table S1

|  |  |  |  |
| --- | --- | --- | --- |
| Sanger sequencing | NEK3: c.805-1G>C | F | 5' TGAAATGACATCACGGCAG 3' |
|  |  | R | 5' TCATTTGAGGAGTATCTCCGA 3' |
|  |  |  |  |
| Sanger sequencing | NEK3: c.1117dupA | F | 5' GAGGAAAGCCAGTTCACCA 3' |
|  |  | R | 5' GTGTGACGCCAAATACCCT 3' |
|  |  |  |  |
| Sanger sequencing | NEK3: c.29T>C | F | 5' GTGACTGCGTGAGTGGAGC 3' |
|  |  | R | 5' TAGTGACCTTGGGAAGCCTTA 3' |
|  |  |  |  |
| Sanger sequencing | NEK3: c.356A>G | F | 5' AGCCAGTTACTGCATTCTTGA 3' |
|  |  | R | 5' TAAACAAAACTGTCCCAAAATG 3' |
|  |  |  |  |
| Splicing test | NEK3 primer 2 | F | 5' CACTGCCGTCTCATTACTCCTA 3' |
|  |  | R | 5' CTTGTTCTTCCTCTTGCACTGT 3' |
|  |  |  |  |
| Splicing Test | NEK3 primer 1 | F | 5' ATGGTGACTGCGTGAGTG 3' |
|  |  | R | 5' GCTAAAAGAACAGCCTCCT 3' |
|  |  |  |  |
| qPCR | NEK3 | F | 5' TGAGAATGATTGGGGAGG 3' |
|  |  | R | 5' GGGTGTTTCATTTTGGCTA 3' |
|  |  |  |  |
| qPCR | NUP205 | F | 5' GATTTTAGAAGTGGGCTGGCT 3' |
|  |  | R | 5' CGTCTGACAAGAGCCTGTATGA 3' |
|  |  |  |  |
| qPCR | GAPDH | F | 5' GAAGGTGAAGGTCGGAGTC 3' |
|  |  | R | 5' GAAGATGGTGATGGGATTTC 3' |
|  |  |  |  |
| qPCR | NNMT | F | 5' CCAAGGACACCTATCTAAGCC 3' |
|  |  | R | 5' AAAGATTTTTCAGAAGGTGCTT 3' |
|  |  |  |  |
| qPCR | SIRT2 | F | 5' CAAGGGCTTCAGCACCG 3' |
|  |  | R | 5' GGGAGAATAAGTTCCGCAG 3' |
|  |  |  |  |
| siRNA#1 | NEK3 targeted sequences |  | GGAATATGGTGAGGAAGTA |
|  |  |  |  |
| siRNA#2 | NEK3 targeted sequences |  | GGAGTAAATCACATTCACA |
|  |  |  |  |
| siRNA#3 | NEK3 targeted sequences |  | GTTGCATCCTGTATGAACT |
|  |  |  |  |
| Ctrl siRNA | Negative control |  | TTCTCCGAACGTGTCACGT |
|  |  |  |  |
| siRNA#1 | NUP205 targeted sequences | | GATGAAGATGCTCGAATGA |
|  |  |  |  |
| siRNA#2 | NUP205 targeted sequences | | CTGGCTTTGACCCTTATTT |
