# Supplementary material for: Biallelic loss of function NEK3 mutations deacetylate α-tubulin and downregulate NUP205 that predispose individuals to cilia-related abnormal cardiac left–right patterning
Source: Cell Death Dis. 2020 Nov 23;11(11):1005. doi: 10.1038/s41419-020-03214-1 (PMC7684299; doi:10.1038/s41419-020-03214-1)
Supplement: Supplementary file 4 — Supplementary Table S2 [file 41419_2020_3214_MOESM4_ESM.docx]

Supplementary Table S2

| Gene name | NEK3 siRNA#1 vs Ctrl | | NEK3 siRNA#2 vs Ctrl | | NEK3 siRNA#3 vs Ctrl | |
| --- | --- | --- | --- | --- | --- | --- |
|  | Log2(fc) | P-adjust valu.adjust | Log2(fc) | P-adjust | Log2(fc) | P-adjust |
| KIF7 | **-1.1948** | 8.50E-05 | **-1.2381** | 5.02E-05 | -0.09402 | 0.8036 |
