# Supplementary material for: Biallelic loss of function NEK3 mutations deacetylate α-tubulin and downregulate NUP205 that predispose individuals to cilia-related abnormal cardiac left–right patterning
Source: Cell Death Dis. 2020 Nov 23;11(11):1005. doi: 10.1038/s41419-020-03214-1 (PMC7684299; doi:10.1038/s41419-020-03214-1)
Supplement: Supplementary file 5 — Supplementary Table S3 [file 41419_2020_3214_MOESM5_ESM.docx]

Supplementary Table S3

| Gene name | NEK3 siRNA#1 vs Ctrl | | NEK3 siRNA#2 vs Ctrl | | NEK3 siRNA#3 vs Ctrl | |
| --- | --- | --- | --- | --- | --- | --- |
|  | Log2(fc) | P-adjust valu.adjust | Log2(fc) | P-adjust | Log2(fc) | P-adjust |
| NUP205 | **-1.58835** | 0.001192 | **-1.4393** | 0.001201 | -0.00731 | 1 |
| NUP155 | **-1.34582** | 0.00922 | **-1.04265** | 0.000818 | -0.15114 | 1 |
| NUP188 | **-1.30955** | 0.017604 | **-1.37381** | 0.015588 | 0.01782 | 1 |
| NUP35 | -0.83774 | 0.019041 | **-1.13603** | 0.00104 | 0.051012 | 1 |
| NUP153 | -0.68311 | 0.020297 | -0.63962 | 0.05172 | -0.11134 | 1 |
| NUP88 | -0.67123 | 0.026718 | -0.6908 | 0.033056 | -0.13175 | 1 |
| NUP93 | -0.66586 | 0.027203 | -0.44276 | 0.272198 | -0.0417 | 1 |
| NUP85 | -0.63571 | 0.045217 | -0.62466 | 0.073328 | -0.00287 | 1 |
| NUP160 | -0.59101 | 0.05653 | -0.73648 | 0.016975 | -0.46921 | 0.664913 |
| NUP210 | -0.58237 | 0.059421 | -1.05416 | 9.73E-05 | 0.268394 | 0.955014 |
| NUP62 | -0.47037 | 0.16252 | -0.78614 | 0.008505 | 0.093166 | 1 |
| NUP37 | -0.48097 | 0.191607 | -0.78689 | 0.016629 | -0.02614 | 1 |
| NUP210L | -1.82597 | 0.223066 | -1.11548 | 0.553638 | 0.894507 | 0.937463 |
| NUP214 | -0.41527 | 0.246407 | -0.30791 | 0.550024 | -0.23095 | 1 |
| NUP43 | -0.38235 | 0.320715 | -0.61471 | 0.074288 | 0.008445 | 1 |
| NUP98 | -0.35908 | 0.342474 | -0.56807 | 0.098071 | 0.0536 | 1 |
| NUP54 | -0.24671 | 0.62866 | -0.43344 | 0.311993 | -0.08705 | 1 |
| NUP133 | -0.01106 | 0.995101 | -0.14187 | 0.922721 | -0.00843 | 1 |
